# Datasets and analyses of molecular dynamics simulations of covalent binary and ternary complexes of MHC class I-related molecule/T-cell receptor (MR1/TCR) agonists to understand complex formation and conditions of fluorescent labelling

**DOI:** 10.1016/j.dib.2020.106704

**Published:** 2020-12-29

**Authors:** Anke Steinmetz, Thomas Yvorra, Pascal Retailleau, Olivier Lantz, Frédéric Schmidt

**Affiliations:** a Centre de Recherche et Développement Vitry-Alfortville, IDD/ISDD, Sanofi-Aventis R&D, Vitry-surSeine, 94400, France; b Institut Curie, PSL University, CNRS UMR3666, INSERM U1143, Paris, 75005, France; c Institut de Chimie des Substances Naturelles, CNRS UPR 2301, Université Paris-Saclay, 1 avenue de la Terrasse, Gif-sur-Yvette, 91190, France; d Institut Curie, PSL University, INSERM U932, Paris, 75005, France; e Institut Curie; Laboratoire d'Immunologie Clinique, Paris, 75005, France; f Centre d'Investigation Clinique en Biothérapie, Institut Curie (CIC-BT1428), Paris, 75005, France

**Keywords:** Molecular dynamics of MAIT cell ligands, Ternary MR1/TCR ligand complexes, Ligand induced-fit, Photoinduced electron transfer (PET) of Alexa Fluor™ 488 fluorophore

## Abstract

Data of molecular dynamics (MD) simulations were obtained for mucosal-associated invariant T (MAIT) cell ligands complexed with MR1 or MR1/TCR. Ligands included in the simulations were natural ligands 5-(2-oxoethylideneamino)-6-D-ribitylaminouracil (5-OE-RU), 5-(2-oxopropylideneamino)-6-(D-ribitylamino)uracil (5-OP-RU), their C5’ ethinylated analogs in S or R configuration, as well as the corresponding fluorophore-reacted products. All-atom models of the binary and ternary complexes were constructed using PDB entry 4NQE and docked poses [1]. Missing loops, N- and C-termini were completed by homology modelling, the loop conformations optimized, and the models energy minimized prior to setup for MD simulations. A standard pre-equilibration protocol was applied before the production phase of 120 ns simulation as NPT ensemble at 300 K and 1 atm applying an explicit solvent model with OPLS3 force field parameters. Atomic coordinates and energies were recorded every 60 ps and 12 ps, respectively. The corresponding raw data files of the MD simulations are part of this dataset. All simulations were analysed with respect to root mean square deviations (rmsd) and root mean square fluctuations (rmsf) of the coordinates of protein and ligand atoms, stability of protein secondary structure, protein-ligand contacts, ligand torsion profiles, and ligand properties. More detailed statistics of non-covalent interaction counts were also collected. Radial distribution functions (rdf) were calculated when relevant. Visualization of the trajectories permits appreciation of the molecular dynamics of both, ligands and proteins and their interactions, thereby supporting drug design of MAIT cell ligands; furthermore, additional analysis of e.g. conformational changes or interactions not reported in the primary publication [1] can be performed on the data. The raw data may also be used as starting point for extension of the simulations or more sophisticated MD techniques.

## Specifications Table

**Table:** 

Subject	Biological Sciences
Specific subject area	Computational molecular biophysics
Type of data	• Raw data of molecular dynamics simulations: Desmond input and output files as generated by the implementation in Maestro molecular modelling package commercialized by Schrödinger Inc.; compressed • Simulation Interactions Diagram Reports in pdf format (SIDR.pdf) • Statistics of non-covalent interaction counts in xlsx format (statistics_ncic.xlsx)
How data were acquired	The data were generated by molecular dynamics (MD) simulations by program Desmond on NVIDIA V100 graphical processing units (GPUs), prepared and submitted via graphical interface Maestro, on a linux workstation with Redhat 7 operating system. These programs were part of Schrödinger software 2020.u1 release.
Data format	• Raw: all raw data in Desmond format as there are input & output coordinates, parameter, command, energy, checkpoint, log, and trajectory files • Analysed: pdf and xlsx
Parameters for data collection	All-atom models with OPLS3.0 forcefield, explicit SPC solvent, NPT ensemble at 300 K & 1 atm, Na^+^ neutralized systems, infinite boundary conditions, Nose-Hoover chain thermostat, Martyna-Tobias-Klein barostat
Description of data collection	Input models were prepared and subsequently subjected to MD simulations following this protocol: 1. Standard pre-equilibration protocol provided by Schrödinger Inc. comprising 1) Brownian Dynamics, 100 ps, NVT at 10 K, small timesteps, restraints on solute heavy atoms 2) MD, 12 ps, 10 K, NVT, restraints on solute heavy atoms 3) MD, 12 ps, 10 K, NPT, restraints on solute heavy atoms 4) MD, 12 ps, NPT at target temperature (300 K) with restraints on solute except hydrogens 5) MD, 24 ps as NPT ensemble at target temperature (300 K), no restraints 2. The production phase covered 120 ns simulation time with frames saved every 60 ps & energies recorded every 12ps.
Data source location	Institution: Sanofi-Aventis R&D / CRVA City/Town/Region: Vitry-sur-Seine / Ile de France Country: France
Data accessibility	Raw data, pdf and xlsx of data analyses are found in: Repository name: Mendeley Data Data identification number: http://dx.doi.org/10.17632/txms2vk6jb.1http://dx.doi.org/10.17632/wwxnsj66ks.1http://dx.doi.org/10.17632/d9byds5hs6.1 http://dx.doi.org/10.17632/kbvs2cdyjg.1http://dx.doi.org/10.17632/tp62zb6rg4.1http://dx.doi.org/10.17632/4d96x29xkp.1 Direct URL to data: https://data.mendeley.com/datasets/txms2vk6jb/1 (MD_MAITcellLigands_i_2020) https://data.mendeley.com/datasets/wwxnsj66ks/1 (MD_MAITcellLigands_ii_2020) https://data.mendeley.com/datasets/d9byds5hs6/1 (MD_MAITcellLigands_iii_2020) https://data.mendeley.com/datasets/kbvs2cdyjg/1 (MD_MAITcellLigands_iv_2020) https://data.mendeley.com/datasets/tp62zb6rg4/1 (MD_MAITcellLigands_v_2020) https://data.mendeley.com/datasets/4d96x29xkp/1 (MD_MAITcellLigands_vi_2020)
Related research article	Thomas Yvorra, Anke Steinmetz, Pascal Retailleau, Olivier Lantz and Frédéric Schmidt “Synthesis, biological evaluation and molecular modelling of new potent clickable analogues of 5-OP-RU for their use as chemical probes for the study of MAIT cell biology”, Eur. J. Med. Chem. In press. DOI 10.1016/j.ejmech.2020.113066 Pre-proof version available at https://www.sciencedirect.com/science/article/abs/pii/S0223523420310382?via%3Dihub

## Value of the Data

• The broadly designed study of MD of receptor complexes of natural MAIT cell ligands, their ethinylated or fluorescence-labelled derivatives generated data that give insights beyond analysis of interactions observed in crystal structures of ternary ligand/MR1/TCR complexes. The data permit a MD weighted appreciation of crystallographically observed interactions. Thus, they may support drug design of MAIT cell ligands. They were notably generated from models of both, binary and ternary complexes of MAIT cell ligands to give insights into the formation of the ternary complexes.
• Researchers or scientists i) working in the field of MAIT cell biology, specialized in structural biology or molecular modelling or particularly interested in MD of ternary ligand/MR1/TCR or binary ligand/MR1 complexes or ii) interested in fluorophore/protein interactions may benefit from access to these data.
• Trajectories can be visualized for appreciation of the molecular dynamics of both, ligands and proteins and their interactions; they can be further used for additional analysis of e.g. conformational changes or interactions not explicitly reported in the primary citation. The MD files can also serve to start extensions of the simulations or as input for more sophisticated MD approaches to support drug design.
• The data provide a first step to the risk assessment of photoinduced electron transfer (PET) in Alexa Fluor^TM^ 488 fluorophore-labelled ligand/MR1 complexes as PET requires at least transient van der Waals contacts of excited donor and suitable acceptor groups which can be assessed by MD simulations [1].

## 1 Data Description

The MD raw data are deposited in Mendeley Data as tar archives. All input and output files required and created by program Desmond as commercialized by Schrodinger Inc. are provided. All files were compressed prior to archiving. The trajectory files found in the directories [MDsimulation_name]_trj have to be decompressed prior to visualization. The 4 simulations of the binary and ternary complexes of 5-OE-RU and 5-OP-RU are found in archive_natural_ligands_5OERU.tar and archive_natural_ligands_5OPRU.tar, respectively. The 4 simulations of the ternary complexes of the R and S stereoisomers of their ethinylated analogues are contained in archive_ethinylated_ligands_SSRR_ternary_2.tar and archive_ethinylated_ligands_SSRS_ternary_2.tar, respectively, while the simulations of the corresponding binary complexes are in archive_ethinylated_ligands_binary.tar. The 4 simulations of the binary complexes of the two stereoisomers of the fluorophore-reacted products are accessible in archive_fluorescent_ligands.tar.

SIDR.pdf contains the Simulation Interactions Diagram Reports in pdf format of all 16 simulations, as well as the plots of radial distribution functions when relevant. The Simulation Interactions Diagram Reports cover analysis of protein-ligand rmsd, protein rmsf, protein secondary structure, ligand rmsf, protein-ligand contacts, ligand torsion profile, and ligand properties.

Statistics_ncic.xlsx reports more detailed statistics of non-covalent interaction counts in xlsx format of all 16 MD simulations.

## 2 Experimental Design, Materials and Methods

MD were simulated for ternary and binary ligand complexes of TCR/MR1 and MR1. Starting models were prepared for the natural ligands 5-OE-RU, 5-OP-RU, their ethinylated analogues in R and S stereochemistry and the corresponding fluorophore-reacted analogues, the latter as binary complexes only. The experimental work accompanied by these simulations only used ligand activation by methylglyoxal [1]. However, both, glyoxal and methylglyoxal activation was assumed for model construction to provide two independent simulations for each ligand probing the interactions with protein and addressing ternary complex formation. Thus, altogether 16 systems subjected to MD simulations generated the data reported here.

Molecular modelling was carried out using Drug Discovery Suite by Schrödinger Inc. in versions 2016.u3 or 2020.u1 to visualize models, design and prepare ligands and protein structures, complete protein models by homology modelling, refine loop conformations, minimize energy, simulate and analyze MD. Thus, programs and tools Maestro, Protein Preparation Wizard, Ligprep, Epik, Glide, PRIME, Macromodel, and Desmond were employed by using standard procedures and settings unless specified otherwise [2], [3], [4], [5], [6], [7]. Force field parameters OPLS3 with implicit or explicit SPC water models were applied [8].

Covalent ternary TCR/MR1 complexes of ethinyl-5-A-RU (**13**) were prepared for molecular dynamics simulations by replacing the original ligand in prepared pdb entry 4NQE [9], chains A, B, G, and H, by selected poses from docking [1], construction of the covalent linkages to K43^A^ assuming ligand activation by either glyoxal or methylglyoxal, and deletion of water molecules sterically interfering with the ligands. Covalent ternary TCR/MR1 complexes of **13** reacted with 3,6-Diamino-9-[2-carboxy-5-carboxamido-(6-azidohexanyl)-phenyl]-4,5-disulfoxanthylium (Alexa TM® 488) fluorophore were prepared from **13** in the docked conformations in the same manner. More precisely, the ethinyl groups were accordingly modified, the fluorophore extensions manually fit to the protein, the covalent linkages constructed, and sterically interfering water molecules deleted. Complexes of 5-OE-RU and 5-OP-RU were prepared from 4NQE. All models were subjected to hydrogen bond optimization and energy minimization of hydrogen atoms in the protein preparation wizard before energy minimization to convergence in Macromodel with a fully flexible zone within 4.5 Å of the ligand, a buffer zone of additional 10 Å, and all other residues frozen while the fillres option was applied. As all published crystallographic models of MR1/TCR complexes lack a few N- or C-terminal residues as well as several short loops, we de novo modelled residues N1^H^ – G3^H^, C-termini V269^A^ – P270^A^, D96^B^ – M99^B^, P198^G^ – S203^G^, E239^H^ – D245^H^, loops L246^A^ – L253^A^, R122^G^ – S130^G^, N176^G^ – D179^G^, and F201^H^ – R210^H^. Thus, homology models of chains A, B, G, and H were built on the energy optimized models using Prime to include missing residues. The inserted residues were subjected to sequential, extended loop refinement in Prime. These models replaced the original chains; chains G and H were omitted in models for simulations of ligand/MR1 binary complexes. Model completion issued a variety of alternative conformations for the missing residues of the 20 completed complexes (SM2 of [1]). As these were bordering crystalline solvent channels, we assumed that conformational variability existed in these regions and accepted all proposed models for molecular dynamics simulations. The novel models including properly formed covalent linkages were again subjected to hydrogen optimization prior to Desmond molecular dynamics setup by neutralizing the systems with sodium ions and simulation box optimization applying a 10 Å buffer zone in all three dimensions.

Molecular dynamics simulations as NPT ensembles were carried out on NVIDIA V100 graphical processing units (GPUs) for 120 ns production phase at 300 K and 1 atm applying Nose-Hoover chain thermostat and Martyna-Tobias-Klein barostat methods. Atomic coordinates (frames) were saved every 60 ps, energy values logged every 12 ps. The production phase was preceded by the standard preequilibration protocol provided with Desmond. They were further analysed by event_analysis.py and analyze_simulation.py scripts provided by Schrödinger. Standard quality and structural analysis showed stable simulations in terms of potential and total energy, volume, temperature, pressure, and secondary structure (SIDR.pdf). In none of the simulations rmsf of the protein chains exceeded 7.0 Å, thus illustrating moderate conformational changes (SIDR.pdf). Rmsf of de novo modelled loops were observed at the same level as crystallographically observed loops. Detailed analysis and precise values of non-covalent intraligand and ligand-protein interactions are reported in supplementary materials SM2 of [1], statistics_ncic.xlsx, and SIDR.pdf. Protein, ligand, and water interactions reported by the analyze_simulation.py script, were summarized by a tailor-made workflow in KNIME version 4.0.2 integrating Schrödinger version 2020.u1 nodes. Rdf of water molecules around the ethinyl or triazole groups were calculated interactively via Maestro and the plots exported as png files. All pdf reports, rdf plots, and KNIME generated tables are available in SIDR.pdf and statistics_ncic.xlsx.

## Ethics Statement

Data generation, acquisition, and analyses did not involve experimentation with human subjects or animals. Neither were data collected from social media platforms.

## CRediT Author Statement

**Anke Steinmetz:** Conceptualization, molecular modelling, MD simulations and their analyses, Knime script, Writing - Original draft preparation, editing, finalization; **Thomas Yvorra, Pascal Retailleau, Olivier Lantz, Frédéric Schmidt**: Writing – Reviewing & co-submission.

## Declaration of Competing Interest

The authors declare that they have no known competing financial interests or personal relationships which have or could be perceived to have influenced the work reported in this article. Else, Anke Steinmetz is an employee of Sanofi-Aventis R&D.
